# Endomembrane architecture and dynamics during secretion of the extracellular matrix of the unicellular charophyte, *Penium margaritaceum*

**DOI:** 10.1093/jxb/eraa039

**Published:** 2020-02-25

**Authors:** David S Domozych, Li Sun, Kattia Palacio-Lopez, Reagan Reed, Susan Jeon, Mingjia Li, Chen Jiao, Iben Sørensen, Zhangjun Fei, Jocelyn K C Rose

**Affiliations:** 1 Department of Biology, Skidmore College, Saratoga Springs, NY, USA; 2 Plant Biology Section, School of Integrative Plant Science, Cornell University, Ithaca, NY, USA; 3 Boyce Thompson Institute, Ithaca, NY, USA; 4 U.S. Department of Agriculture-Agricultural Research Service, Robert W. Holley Center for Agriculture and Health, Ithaca, NY, USA; 5 University of Innsbruck, Austria

**Keywords:** Charophyte, endomembrane system, extracellular matrix, Golgi body, tomography, *Penium*

## Abstract

The extracellular matrix (ECM) of many charophytes, the assemblage of green algae that are the sister group to land plants, is complex, produced in large amounts, and has multiple essential functions. An extensive secretory apparatus and endomembrane system are presumably needed to synthesize and secrete the ECM, but structural details of such a system have not been fully characterized. *Penium margaritaceum* is a valuable unicellular model charophyte for studying secretion dynamics. We report that *Penium* has a highly organized endomembrane system, consisting of 150–200 non-mobile Golgi bodies that process and package ECM components into different sets of vesicles that traffic to the cortical cytoplasm, where they are transported around the cell by cytoplasmic streaming. At either fixed or transient areas, specific cytoplasmic vesicles fuse with the plasma membrane and secrete their constituents. Extracellular polysaccharide (EPS) production was observed to occur in one location of the Golgi body and sometimes in unique Golgi hybrids. Treatment of cells with brefeldin A caused disruption of the Golgi body, and inhibition of EPS secretion and cell wall expansion. The structure of the endomembrane system in *Penium* provides mechanistic insights into how extant charophytes generate large quantities of ECM, which in their ancestors facilitated the colonization of land.

## Teaser

A layered endomembrane system consisting of linear rows of Golgi bodies and a peripheral vesicle network fuel the secretion of the complex extracellular matrix of the unicellular charophyte, *Penium*.

## Introduction

The plant cell extracellular matrix (ECM) is a highly complex covering that forms the interface between the living protoplast and the external environment. The essential functions provided by the ECM are diverse and include controlling cell expansion and morphogenesis, serving as a rigid edifice for defense and support of extensive growth, contributing to absorption and transport of water and solutes, responding to biotic and abiotic stresses, and creating the structural scaffold and communication conduit with other cells (Lionetti and Metraux, 2014; Bidhendi and Geitmann, 2016). The ECM can be considered as two broad interactive domains: the cell wall and an array of materials deposited onto or beyond the cell wall. The cell wall is a fiber-reinforced hydrogel (Ali and Traas, 2016) consisting of crystalline cellulose microfibrils that are embedded in a hydrated assembly of pectic polymers, hemicelluloses, and proteoglycans. These components form distinct microarchitectural networks that often modulate during cell development and in response to external stresses. The second ECM component consists of an assortment of gel-like polysaccharides/proteoglycans and hydrophobic materials that are deposited either onto or in the architecture of the wall. These ECM components have diverse functions that include providing a hydrated matrix for embryo growth during seed germination (Francoz *et al.*, 2015, serving as lubricants for root growth in soils (Iijima *et al.*, 2004) and providing waterproof coatings on various tissues and organs (e.g. cuticles; Dominguez *et al*., 2011; Yeats and Rose, 2013; Niklas *et al.*, 2017). Clearly, the ECM is of central importance to plant life, and the biosynthesis and secretion of the ECM both requires substantial amounts of photosynthetically fixed carbon and involves a significant portion of the genetic machinery of the cell (Popper *et al.*, 2011).

Much of the endomembrane system and membrane trafficking networks are devoted to the biosynthesis and deposition of the ECM components (Kim and Brandizzi, 2016; van de Meene *et al.*, 2017; Lampugnani *et al.*, 2018). Endomembrane dynamics during ECM production are organized around its constituent compartments, including the endoplasmic reticulum (ER), the Golgi apparatus (GA), the *trans*-Golgi network (TGN), pre-vacuolar compartments, an assortment of vesicles, and the vacuolar network. Communication and interaction between these membranous compartments are mediated by numerous proteins (e.g. COPI, COPII, SNAREs, RAB, and ROP GTPases) that are organized into highly complex signaling networks (Gendre *et al.*, 2013; Kim and Brandizzi, 2014, 2016). These work in close spatiotemporal synchrony with the cytoskeletal network and plasma membrane (Szymanski and Cosgrove, 2009; Brandizzi and Wastenys, 2013; Oda, 2015; Cao *et al.*, 2016; Elliott and Shaw, 2018). Much has been learned about endomembrane architecture and membrane trafficking dynamics in relation to ECM biosynthesis in select land plants (van de Meene *et al.*, 2017). This information is providing critical insight into the subcellular dynamics of plant cells, the cellular responses to external stresses imposed by terrestrial ecosystems (e.g. desiccation or UV irradiance), and those processes responsible for production of many economically important plant products (e.g. food, wood, paper, biofuels, and pharmaceuticals).

Over 500 million years ago, land plants arose from a charophycean green algal ancestor (i.e. Charophycean Green Algae or basal Streptophyta; Becker and Marin, 2009; Umen, 2014; Delwiche and Cooper, 2015; Domozych *et al.*, 2017*b*; Rensing, 2018). Recent studies have shown that the late divergent charophyte taxon, the Zygnematophyceae, is most probably the sister group to land plants (deVries and Archibald, 2018). The invasion of land by charophytes and subsequent evolutionary events leading to land plants profoundly changed the biogeochemistry of the planet and has resulted in the rich diversity of modern-day flora. Biochemical and immunological studies have shown that the cell walls of late divergent charophytes (i.e. Zygnematophyceae, Charophyceae, and Coleochaetophyceae) have polymer profiles notably similar to those found in cell walls of many land plants (Sørensen *et al.*, 2011; Domozych *et al*., 2012, 2014). This has led to the supposition that pre-adaptation of cell walls was critical for emergence onto, and successful colonization of, terrestrial habitats. The ECM of zygnematophycean taxa also consists of mucilage- or gel-like extracellular polysaccharides (EPS) that are often produced in prodigious amounts and secreted beyond the wall. The EPS functions in gliding motility, anti-desiccation, salt tolerance, and in providing the architectural platform for communication with other microorganisms (Boney, 1981; Oertel *et al.*, 2004; Kiemle *et al.*, 2007; Domozych and Domozych, 2008; Torn *et al.*, 2014). These multiple functions are critical for algae living in transient shallow wetlands that have to rapidly adapt to stresses from both aquatic and terrestrial sources. Biosynthesis and secretion of these diverse ECM components require an extensive endomembrane system and membrane trafficking networks that are highly responsive to external stimuli (Lütz-Meindl and Brosch-Salomon, 2000; Holzinger *et al*., 2000, 2002; Oertel *et al.*, 2004; Eder *et al.*, 2008; Eder and Lütz-Meindl, 2010; Foissner and Wastenys, 2012; Holzinger and Karsten, 2013; Foissner *et al.*, 2016; Lütz-Meindl, 2016). However, the detailed structure of the specific endomembrane components, their location, and dynamic interactions during cell development and their roles in the biosynthesis/deposition of specific ECM components are mostly unresolved. Elucidation of these phenomena would both advance understanding of endomembrane structure/function in plant cells and provide insight into ECM biosynthesis and secretion dynamics that were critical for invasion and exploitation of terrestrial habitats by ancient charophytes.

The unicellular zygnematophycean alga, *Penium margaritaceum*, has become an important organism for studying ECM structure and deposition. Previous studies have characterized the distinct pectin-rich cell wall (Sørensen *et al*, 2011; Domozych *et al.*, 2014), EPS gel (Domozych *et al.*, 2005), and secretome (Ruiz-May *et al.*, 2018) of *Penium.* Moreover, its relatively fast growth rate, and amenability to experimental treatment and interrogation, including a range of microscopy technologies (Rydahl *et al.*, 2015; Domozych *et al.*, 2017*a*), make it an attractive organism for analyses of endomembrane dynamics during ECM secretion. In this study, we provide details of the endomembrane system architecture and secretory trafficking networks of *Penium*. Using data derived from multiple microscopy-based techniques in combination with experimental manipulation, we provide a model of the ECM biosynthetic machinery.

## Materials and methods

### Algal material

*Penium margaritaceum* Brébisson (Skidmore College Algal Culture Collection, clone Skd#8) was maintained in sterile liquid cultures of Woods Hole Medium (Domozych *et al.*, 2017*a*) supplemented with soil extract (WHS), pH 7.2 at 18±3 °C in a photoperiod of 16 h light/8 h dark with 74 μmol photons m^–2^ s^–1^ white fluorescent light. The cells were subcultured every week, and cells from log-phase cultures (7- to 14-day-old cultures) were used for the experiments. For high light treatments, cells were cultured for 14 d under a light intensity of 200 μmol photons m^–2^ s^–1^ of cool white fluorescent light. For desiccation studies, cells were cultured for 2 weeks on sterile cellophane sheets (ThermoFisher) placed on WHS solidified with 2% agarose.

### Labeling with intracellular probes and monoclonal antibodies

All chemicals were obtained from either Sigma-Aldrich (St. Louis, MO, USA) or Molecular Probes^®^ (Eugene, OR, USA). Live cell labeling was performed at room temperature, in the dark, and with constant gentle shaking. Cells were collected and thoroughly washed to remove residual EPS from the cell surface that might interfere with labeling. Washed cells were then incubated in fresh WHS medium containing 4.4 µM 5(6)-CFDA (5,6-carboxyfluorescein diacetate) for 15 min, 1.7 µM DiOC_6_(3) (3,3'-dihexyloxacarbocyanine iodide), 2 µM Lyso Tracker™ Red, or 1.25 µM yeast vacuole membrane marker MDY-64 (aminonaphthylethenylpyridinium) for 30 min in the dark and subsequently washed three times with WHS prior to imaging by microscopy. Microscopic observation was performed immediately from min 1 to min 60 after adding the dye. For rhodamine–phalloidin or anti-tubulin labeling, cells were processed using previously described techniques (Ochs *et al.*, 2014).

For assessment of wall and cell expansion under experimental conditions, samples of treated cells were labeled with JIM5 monoclonal antibodies (mAbs) as described in Domozych *et al.* (2014). For assessment of EPS secretion, 50 μl cell samples were gently removed from culture and placed on a glass slide. A 50 μl aliquot of 0.5% 0.75 μm Fluoresbrite beads (Warrington, PA, USA) was gently added to the drop, and after 5 min the cells were observed with fluorescence light microscopy (FLM).

### Experimental analysis

Cells were collected from 1-week-old liquid cultures and washed three times in WHS. They were then incubated in one of the following agents: 1 μg ml^–1^ brefeldin A (BFA; Sigma), 1 μM amiprophos-methyl (APM; Sigma), μg ml^–1^ cytochalasin B (CB), and 5 μg ml^–1^ latrunculin B (LatB). Samples were processed for labeling or fixation after 1, 8, 24, 48, and 72 h. Controls were prepared by culturing cells in 0.1% ethanol or DMSO in WHS for comparable time periods. These two solvents were used for making the stock solutions of the specific subcellular agents. For each agent tested and the control, 50 cells from each of three replicate cultures were sampled and measured. For reversibility experiments, treated cells were collected, washed three times with WHS, and placed back in culture. Samples were removed and processed for labeling or TEM after 1, 4, 8, 24, 48, and 72 h.

### Differential interference contrast microscopy (DIC), FLM, and confocal laser scanning microscopy (CLSM)

DIC and FLM images were taken with either an Olympus BX-63 or BX-60 light microscope. CLSM images were taken with an Olympus Fluoview 1200 CLSM. DIC images were obtained using a U Plan Apo ×100 lens. FLM and CLSM images were obtained with a U Plan Apo ×60 lens with fluorescein isothiocyanate (FITC) or tetramethylrhodamine isothiocyanate (TRITC) filter sets.

### TEM

Cells were prepared for TEM using freeze spraying techniques described in Rydahl *et al.* (2015). Immunogold labeling of thin sections employed mAbs from Plant Probes, Leeds, UK (JIM13 and JIM5; http://www.plantprobes.net), the Complex Carbohydrate Research Center (CCRC-M80) of the University of Georgia, USA, or from Dr Marie-Christine Ralet (INRA-RU) of INRA, Nantes, France. Immunogold-labeled and regular sections were imaged using a Zeiss Libra 120 transmission electron microscope.

### Electron tomography

Serial sections (150 nm section thickness) of cryo-fixed and embedded *Penium* samples were collected on formvar-coated copper slot grids. Each of four serial sections was tilted from –60° to +60° and the tilting series images were taken with increments at ×8000 with the 120 kV transmission electron microscope. To obtain data for a dual-axis tomogram, the orthogonal tilt serials with the same tilt angle range and increments were collected after the sample grid was manually rotated 90° in the sample rod. The images were captured by an Olympus Cantega G2 digital camera that covers an area of 3.8×3.8 µm^2^ and has a resolution of 2048×2048 pixels, at a pixel size of 1.859 nm. The image focus, tilt, alignment, and capture were controlled by WinTEM software (Zeiss, Germany).

For each serial section, there are two sets of image stacks rotated around two orthogonal axes. Each section’s single-axis tomograms were reconstructed by the Etomo software interface in the IMOD software package (https://bio3d.colorado.edu/imod/). Two orthogonal single-axis tomograms were merged into one with a warping procedure (Mastronarde, 1997). The above process was repeated for all four sections. Then the four section tomograms are joined by manually aligning larger structures from the bottom of one tomogram to the top of the adjacent tomogram using Midas in the IMOD software. The tomogram join was then refined by fiducial markers on trajectory features though the adjacent tomograms (Mastronarde *et al.*, 1992).

After completing the tomogram reconstruction, the three-dimensional model of the joined tomogram was built in the 3dmod software interface, which we refer to as ‘modeling’. In modeling, each layer of the Golgi stack was considered as an individual ‘object’, which was assigned in different colors. Free vesicles around the Golgi stack were modeled by a single sphere in that position. The *cis* face was identified as that being closest to the underlying ER, the *trans* face being at the opposite pole from the *cis* face and the medial zone consisting of cisternae in between both faces. These zones and associated vesicles were distinguished in our model via different colors. Finally, after all the contours have been drawn on all the consecutive tomogram slices, imod mesh, which uses a mesh triangular computation to define the surface of each object, was applied to achieve a smooth three-dimensional surface for each object. Due to the initial exposure to the electron beam causing some degree of section collapse, the *z*-dimension of our model was stretched by a factor of 1.47 for accurately displaying the model.

### BLAST search

We used amino acid sequences of genes with known functions in *Arabidopsis thaliana* as queries in a local BLAST search (<1e-5) through our in-house *Penium* protein database.

## Results

### General architecture of the cell

**Fig. 1.**
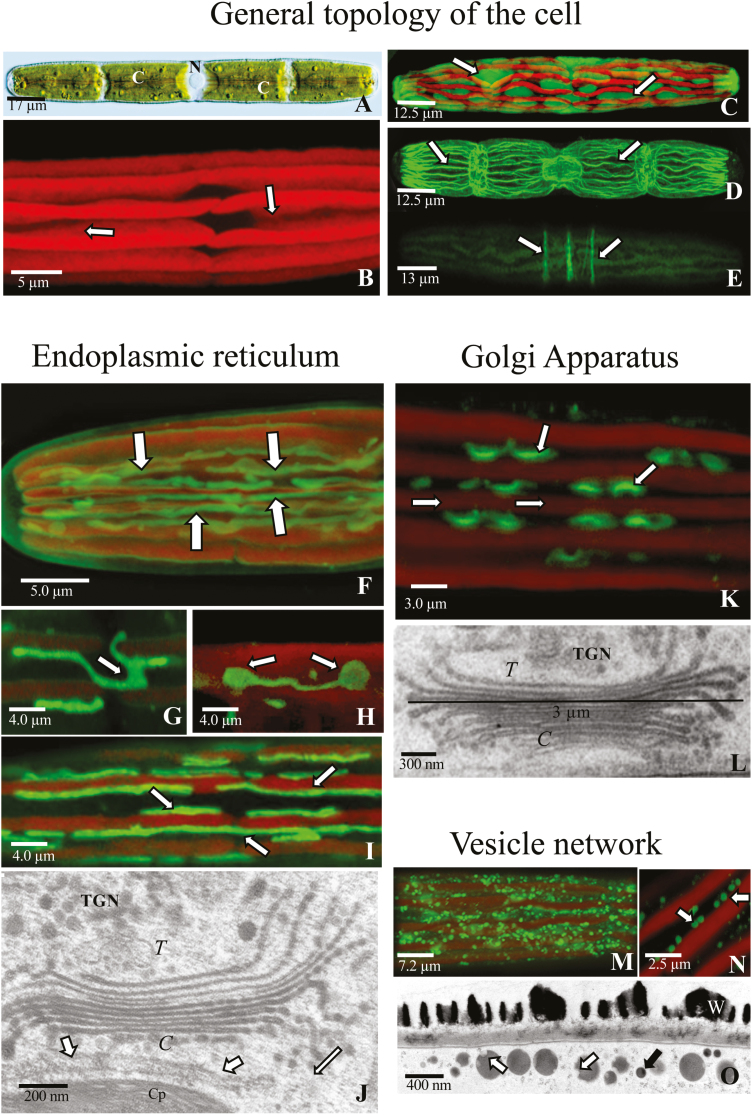
General topology of *Penium* as shown by differential interference contrast microscopy (A), confocal laser scanning microscopy (CLSM) (B–I), (K), (M), and (N), and TEM (J), (L), and (O). (A) A typical *Penium* cell highlighting its cylindrical shape, its nucleus (N) situated in the central isthmus zone and 2–4 chloroplasts (C). (B) Chlorophyll autofluorescence of the multilobed chloroplasts and the associated narrow cytoplasmic valleys (arrows). (C) CFDA labeling of the cytoplasm (arrows) in the valleys between the chloroplast lobes (in green). (D) Rhodamine–phalloidin labeling highlighting actin bundles (arrows) in the peripheral cytoplasm. (E) Anti-tubulin labeling revealing bands of microtubules (arrows) at the isthmus zone. (F) DIOC_6_(3) labeling of the endoplasmic reticulum (ER) (arrows) found in the cytoplasmic channels. (G) DIOC_6_(3) labeling showing the branched nature of the ER (arrow). (H) DIOC_6_(3) labeling showing the flattening of ER tubules into plate-like structures (arrow). (I) DIOC_6_(3) labeling revealing the close physical proximity of the ER (arrows) to the chloroplast (red). (J) The close proximity of the ER (arrows) to the chloroplast envelope (Cp) and *cis* (*C*) face of a Golgi body. The *trans* face (*T*) and *trans-*Golgi network (TGN) are also apparent. (K) MDY-64 labeling of Golgi bodies (arrows) found in cytoplasmic valleys created by the chloroplast lobes (red). (L) Golgi body showing the distinct *cis* (*C*), *trans* (*T*), and TGN regions. Golgi bodies measure 2–3 μm from cisternal stack edge to edge (line). (M) Lysotracker labeling of a pool of large vesicles (green) in the cortical cytoplasm. (N) Lysotracker labeling of the large fluorescent vesicles that occasionally contain an unstained punctate center (arrows). (O) The cortical cytoplasm revealing both large (white arrows) EPS-carrying vesicles and small (black arrow) vesicles. Note the unlabeled zones in several of the large vesicles.

*Penium margaritaceum* (Ehrenberg; *Brebisson*) is a unicellular desmid exhibiting a simple cylindroid shape with rounded poles (Fig. 1A). Each cell measures 17 μm in width at the cell center, or isthmus, and cell length varies from 125 μm to 225 μm. The nucleus resides in the isthmus, and this central zone serves as the site of cell and cell wall expansion and division. In the center of each of the two semi-cells are 1–2 multilobed chloroplasts. The lobes create deep cytoplasmic valleys that run the length of each semi-cell (Fig. 1B, C) that contain organized networks of organelles, including highly elongated and branched mitochondria (Supplementary Fig. S2A, B at *JXB* online) and much of the endomembrane system. The cortical cytoplasm forms a thin (300–500 nm) layer adjacent to the plasma membrane, displays rapid cytoplasmic streaming, and contains multiple parallel bundles of actin microfilaments (Fig. 1D). Bands of microtubules running perpendicular to the long axis of the cell were present in the cortical cytoplasm of the isthmus zone (Fig. 1E; see also Ochs *et al.*, 2014).

### Endoplasmic reticulum and Golgi apparatus

In order to observe specific components of the endomembrane system, we labeled live cells with organelle-specific markers, before imaging the labeling patterns with CLSM and correlating them with ultrastructural data derived from TEM imaging. For fluorescence imaging of the ER, we used DIOC_6_(3), a lipophilic, cationic, green fluorescent stain that has been used to label the ER in land plants (Sabnis *et al.*, 1997) and charophytes (Klima and Foisnner, 2011). DIOC_6_(3) labels long, and often flattened, tubules of ER that run parallel to the long axis of the cell (i.e. the length of either a semi-cell or a whole cell; Fig. 1F) and are situated ~1–2 μm above the base of each cytoplasmic valley. The ER occasionally branches (Fig. 1G) and often flattens out to yield plate-like cisternae/sheets (Fig. 1H). The ER also forms tight physical associations with lobes of the underlying chloroplast (Fig. 1I). This was confirmed by TEM imaging that reveals the close proximity of the ER to the chloroplast, as well as to the *cis* face of Golgi bodies (Fig. 1J).

We also labeled cells with MDY-64, a hydrophobic styryl dye that has been used to label vacuoles in yeasts and plants (Cole *et al.*, 1998; Wiltshire and Collings, 2009; Scheuring *et al.*, 2015; Huang *et al.*, 2016; Schreiber *et al.* 2017). When cells were incubated in medium containing 1.25 μM MDY-64 for 30 min, rows of slightly curved fluorescent structures were observed in the cytoplasmic valleys ~3–4 μm from the plasma membrane (Fig. 1K). Each of these structures measured between 2.5 μm and 3.0 μm, with an interspacing of 1–2 μm. Based on comparative analysis with TEM longitudinal section profiles (Fig. 1L), we conclude that MDY-64-labeled structures are indeed Golgi bodies. Since MDY-64 is non-toxic and retains a strong fluorescent signaling lifetime, we were able to examine 50 labeled log phase cells using CLSM and determined that there were between 150 and 200 Golgi bodies per cell.

We used Lysotracker to label a population of large vesicles that were carried in the cortical cytoplasm by cytoplasmic streaming (Fig. 1M). CLSM imaging shows that these vesicles are highly fluorescent and sometimes contain a small, non-labeled puncta (Fig. 1N). These 300–400 nm vesicles matched the size of large EPS-carrying vesicles revealed by TEM that have solid, electron-dense cores that are occasionally interrupted by small, non-labeled zones (Fig. 1O). Both these and other smaller vesicles moved rapidly in narrow streaming channels of the peripheral cytoplasm.

### TEM

**Fig. 2.**
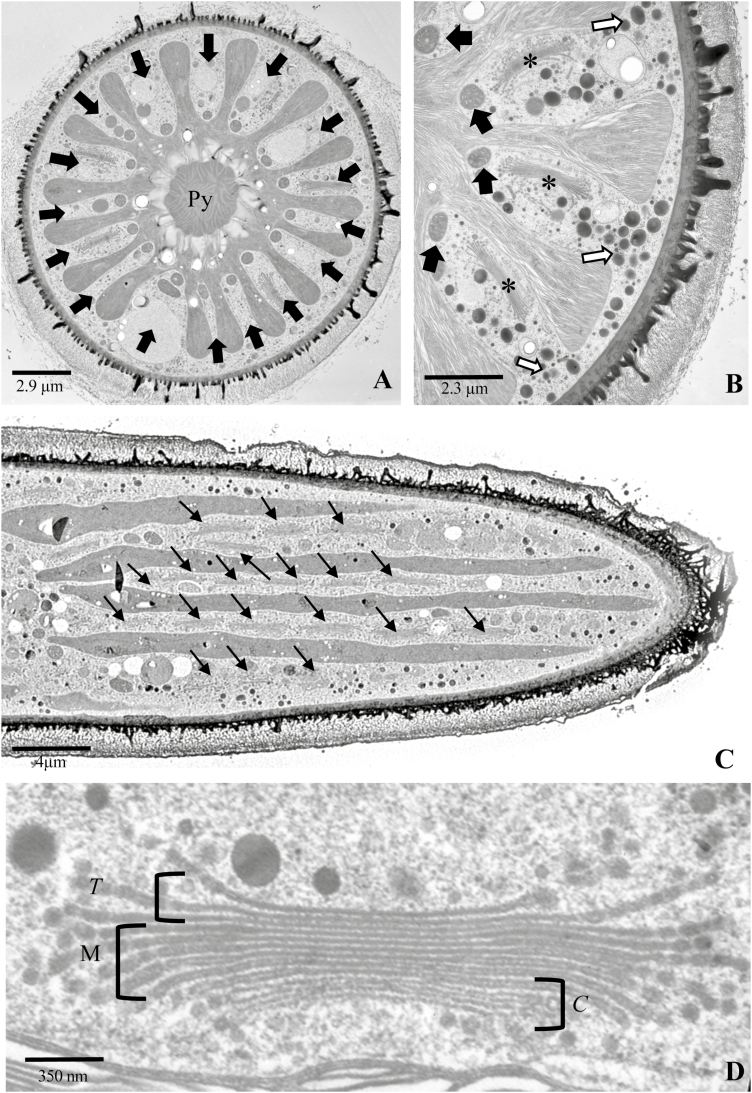
The ultrastructure of the cell and endomembrane architecture. (A) Cell cross-section. The multilobed chloroplast with a pyrenoid (Py) and 16 cytoplasmic valleys (arrows), in which the ER and Golgi are positioned. (B) Magnified view of cytoplasmic valleys, each containing a mitochondrion (black arrows) at the base. Golgi bodies (*) positioned in the mid-valley regions and Golgi-derived vesicles (white arrows) in the cortical region. (C) Longitudinal view of a cell showing Golgi bodies (arrows) forming long networks in the cytoplasmic valleys. (D) Golgi body architecture, with a stack of 12–15 cisternae. The *cis* face cisternae (*C*) have wide unstained (i.e. little or osmium binding or electron density) lumens. The medial (M) and *trans* (*T*) face cisternae have narrow stained (notable osmium labeling) lumens.

TEM imaging provided ultrastructural detail of the subcellular architecture of the endomembrane system and served as a correlative tool to complement the FLM and CLSM imaging of live cells. Imaging of cross-sections reveals 16 cytoplasmic valleys with an average depth of 5–6 μm (measured from the base of a valley to the cortical cytoplasm; Fig. 2A). Organelle positioning is notably stratified, with mitochondria occupying the base of each valley (Supplementary Fig. S2A, B), the ER and GA residing in the mid-valley zones, and vesicles filling the cortical cytoplasm (Fig. 2B). Imaging of longitudinal sections reveals long rows of Golgi bodies in the cytoplasmic valleys (Fig. 2C), with a positioning and size matching that seen with MDY-64 labeling of live cells. Analysis of 25 serial longitudinal sections indicates 150–200 Golgi bodies in each cell. Each Golgi body is ~3.0–3.5 μm across and 1–2 μm from the *cis* to *trans* faces. The 12–15 cisternae are tightly stacked in the center of the Golgi body, while the cisternal peripheries are more loosely bound (Fig. 2D). The luminal spaces of the first 2–3 *cis* face cisternae have variable diameters and display little, if any, electron-dense staining, whereas the lumens of the medial–*trans* face cisternae are compact, with some noticeable staining.

**Fig. 3.**
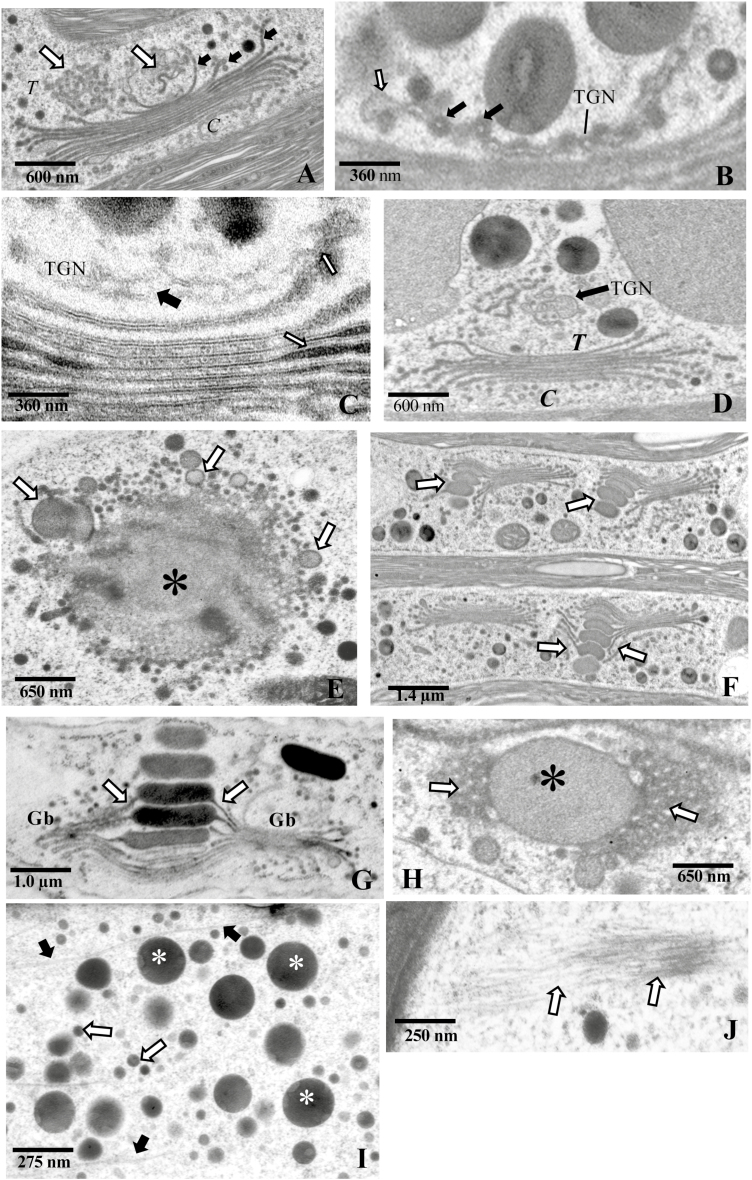
TEM images showing ultrastructure of the endomembrane system. (A) The *trans*-Golgi network (TGN; white arrows) located at the *trans* face (*T*) of the Golgi body. The TGN appear to be derived from terminal *trans* cisternae that curl inward (black arrows). The *cis* (*C*) face is also apparent. (B) Magnified view of the TGN, with multiple coated (black arrows) and uncoated blebs (white arrows). (C) Magnified view of the medial–*trans* region of the cisternal stack. The electron-dense labeling of the peripheries (white arrows) are indicated. The TGN did not contain electron-dense label and had an open lumen (black arrow). (D) TGN removed from the *trans* face (*T*) of the Golgi body. The *cis* face (C) was also apparent. Scale bar=600 nm. (E) Surface view of a *trans* face cisterna (*). Swellings that are destined to bleb off as vesicles on the peripheral edges (arrows) of the cisterna are highlighted. (F) Emergence of large extracellular polysaccharide (EPS) vesicles (arrows) from one side of the Golgi bodies. (G) Magnified image of two Golgi bodies (Gb) in a cell producing EPS. The cisternae of the individual Golgi bodies are attached (arrows). (H) Surface view of cisternae from two different Golgi bodies (arrows) attached via a large vesicle (*). (I) The cortical cytoplasm showing large EPS vesicles (*), smaller vesicles (white arrows), and the interspersed microfilaments (black arrows) in the cortical cytoplasm. (J) Microfilament bundles (arrows) in the cortical cytoplasm.

At the *trans* face, the terminal cisternae curl inward and away from the Golgi stack and form a highly fenestrated TGN (Fig. 3A). The TGN contain both smooth and coated membranous projections (Fig. 3B) and a larger luminal space when compared with the *trans* face cisternae (Fig. 5C). The TGN is typically situated immediately adjacent to the *trans* face, but, in some cases, TGNs are found several microns removed from the *trans* face (Fig. 3D). No TGN or TGN-like compartments are observed in the cortical cytoplasm. Sections through the cisternal face of the medial region of a Golgi body typically reveal a solid center with highly fenestrated edges (Fig. 3E). We conclude that the localized swellings at the cisternal peripheries of the medial region–*tran*s face–TGN (Fig. 3F) yield the EPS-containing vesicles.

Three surprising observations were noted during our ultrastructural studies. First, after analyzing >100 images taken from sections of 50 different cells, we conclude that the large EPS vesicles arise from only one ‘side’ of a Golgi body (Fig. 3F; Supplementary Fig. S2C). Secondly, not all Golgi bodies produce EPS vesicles at the same time. Thirdly, the peripheral swellings of the medial–*trans* cisternae of Golgi bodies that are actively processing EPS are often physically connected to the swellings of an adjacent Golgi body. (Fig. 3G, H). Cisternal fusion is also noted at the *cis* face region where the EPS vesicles are not being produced. It is possible that these profiles represent Golgi body expansion and division occurring coincidentally during EPS production. However, this appears quite different from typical Golgi body division that initiates at the *cis* face and proceeds to the medial and *trans* loci (Supplementary Fig. S2D).

A large and diverse pool of vesicle types is found in the cortical cytoplasm (Fig. 3I), where they are transported around the cell by rapid cytoplasmic streaming as observed by both FLM of MDY64-labeled cells and DIC. This zone is highlighted by networks of single microfilaments and microfilament bundles (Fig. 3I, J). Some of the small wall precursor-containing vesicles are found to associate with the microtubular bands at the isthmus zone (Supplementary Fig. S2E). These vesicles most probably fuse with the plasma membrane at the isthmus and secrete their contents at this site of wall expansion (Supplementary Fig. S2F), while the large EPS-containing vesicles are seen to fuse with the plasma membrane at various points at the cell surface (Supplementary Fig. S2G).

### Electron tomography

**Fig. 4.**
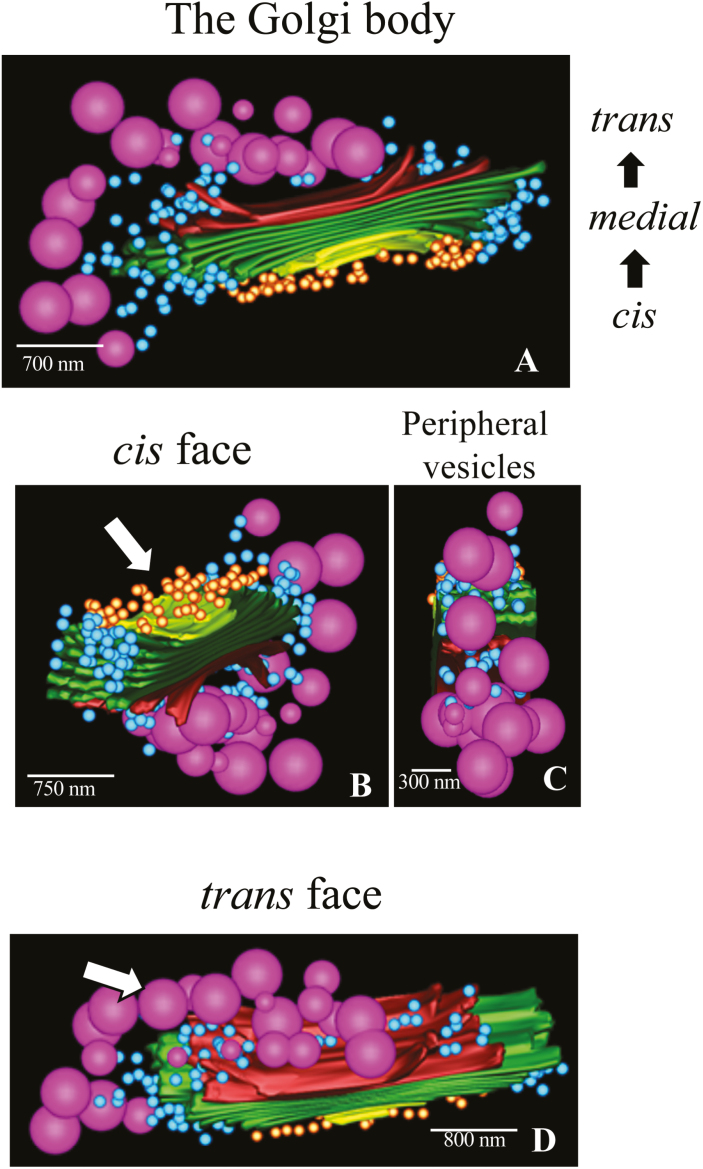
Electron tomography of the Golgi body. (A) Overview of a Golgi body. The Golgi body is surrounded by pools of small vesicles at the cisternal peripheries (blue) and at the *cis* face (yellow vesicles). Large extracellular polysaccharide (EPS) vesicles are present at the post-*trans* region and on one side (purple). Note that the EPS vesicles are found on one side of the Golgi body. (B) View of the *cis* face (arrow) showing the small vesicles (yellow) at the pre-*cis* area. These vesicles are transition vesicles that interface the Golgi body with the ER. (C) View of the periphery of a Golgi body producing large vesicles. (D) View of the *trans* face (arrow) showing the large number of large and small vesicles.

Electron tomography was employed in order to provide three-dimensional profiles of Golgi body structure. It was seen that the Golgi body displays notable polarity, with clearly distinguishable *cis*, medial, and *trans* regions (Fig. 4A), and that several populations of vesicles surround cisternal stacks. Small vesicles fill the zone immediately near the *cis* face (Fig. 4B), while large EPS vesicles are at the post-*trans* face region, but more importantly only on one side or region of the cisternal stack (Fig. 4B–D). These are interspersed with multiple small vesicles (Fig. 4D).

### Immunogold labeling: wall polymer and EPS processing

**Fig. 5.**
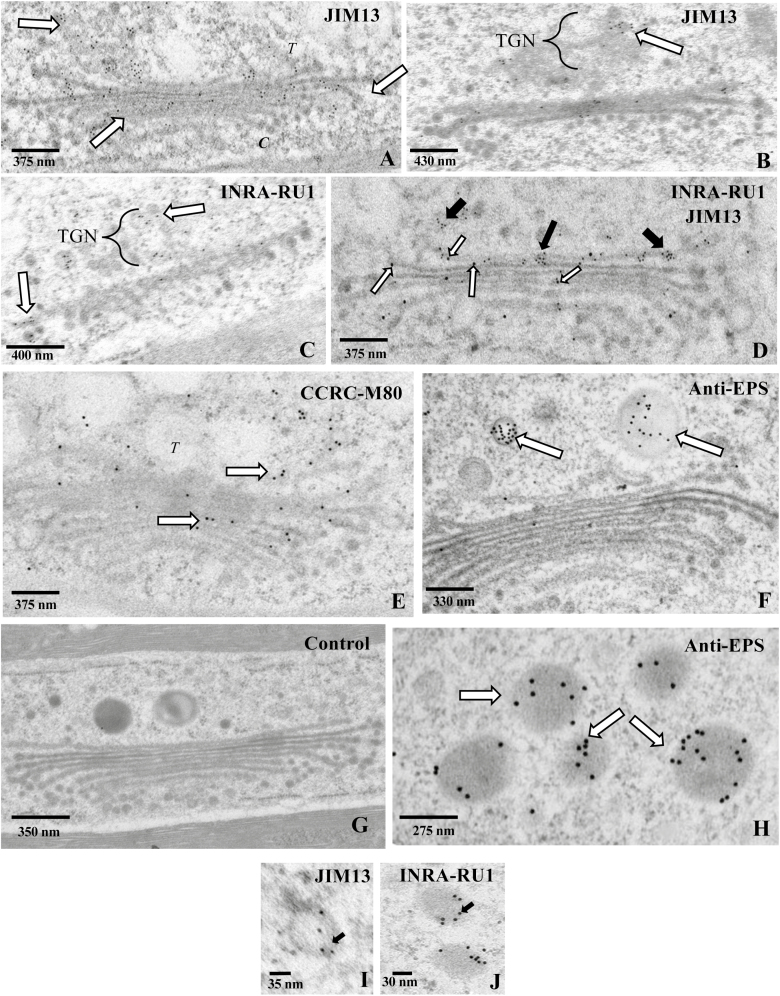
Immunogold labeling and TEM of the Golgi body. (A) Labeling of the Golgi body with an anti-arabinogalactan protein (AGP) mAb, JIM13. The label is seen throughout the Golgi, with a greater concentration of gold particles (black particles or arrows) at the *trans* face (*T*). The *cis* face (*C*) is also apparent. (B) JIM13 labeling of the *trans*-Golgi network (TGN, arrow). (C) Anti-rhamnogalacturonan (RG)-I mAb, INRA-RU1, labeling (arrows) of the cisternal peripheries and TGN. (D) Co-labeling of a Golgi body with JIM13 (black arrows) and INRA-RU1 (white arrows). (E) Anti-RG-I labeling with the CCRC-M80 mAb. The label (arrows) is more prevalent at the *trans* face (*T*). (F) Anti- extracellular polysaccharide (EPS) labeling of large Golgi-derived vesicles (arrows). (G) Control (primary antibody was omitted). (H) Anti-EPS labeling of large vesicles (arrows) in the cortical cytoplasm. (I) JIM13 labeling (arrow) of a small vesicle in the cortical cytoplasm. (J) INRA-RU1 labeling (arrow) of small vesicles in the cortical cytoplasm.

Immunogold labeling with antibodies that recognize ECM components was employed to identify ECM processing (i.e. biosynthesis and packing) sites in the endomembrane system. Sections of cryo-fixed/non-osmicated cells were labeled with various mAbs with binding specificities for particular plant cell wall polymer epitopes or the EPS. These include: (i) the mAb, JIM13, with specificity for β-d-glucuronic acid (GlcA)-(1–3)-α-d-galacturonic acid (GalA)-(1,2)-α-l-rhamnose (Rha), which is often associated with arabinogalactan proteins (AGPs) (Knox *et al.*, 1991); (ii) the rhamnogalacturonan I (RG-I)-binding mAb, INRA-RU1, with specificity for Rha-(1,4)-GalA-(1,2)-Rha-(1,4)-GalA-(1–2), which makes up the backbone of RG-I (Ralet *et al.*, 2010); (iii) CCRC-M80, with specificity for RG-I (Pattathil *et al.*, 2010); and (iv) an EPS-binding polyclonal antibody (Skd-108). In the ECM, JIM13 labels a network of fine fibrils on the outer part of the cell wall (Supplementary Fig. S3A), INRA-RU1 labels the medial region of the cell wall (Supplementary Fig. S3B), CCRCM-80 labels the plasma membrane, and the anti-EPS antibody labels the EPS (Domozych *et al.*, 2005). JIM13 labels the cisternal peripheries (Fig. 5A) and the TGN (Fig. 5B) of the Golgi body. INRA-RU1 also labels medial to *trans* Golgi cisternae and the TGN (Fig. 5C). In co-labeling experiments, both JIM13 and INRA-RUI label the same Golgi bodies (Fig. 5D). CCRCM-80 labels medial–*trans* face cisternae and the TGN (Fig. 5E). Anti-EPS labeling is located in large swellings and vesicles at the *trans* face region (Fig. 5F). Control experiments included elimination of the primary antibody and revealed no labeling (Fig. 5G). We also examined labeling of vesicles with the various antibodies. Anti-EPS labels the large 300–400 nm vesicles (Fig. 5H), while small 50–100 nm vesicles were labeled with JIM13 and INRA-RUI (Fig. 5I, J). Based on these observations, we then consider the large vesicles as EPS-containing vesicles and the smaller vesicles as wall precursor-containing vesicles. We observed no vesicles that were co-labeled with acombination of antibodies.

### Experimental analysis of the ER and Golgi apparatus with subcellular-disrupting agents

**Fig. 6.**
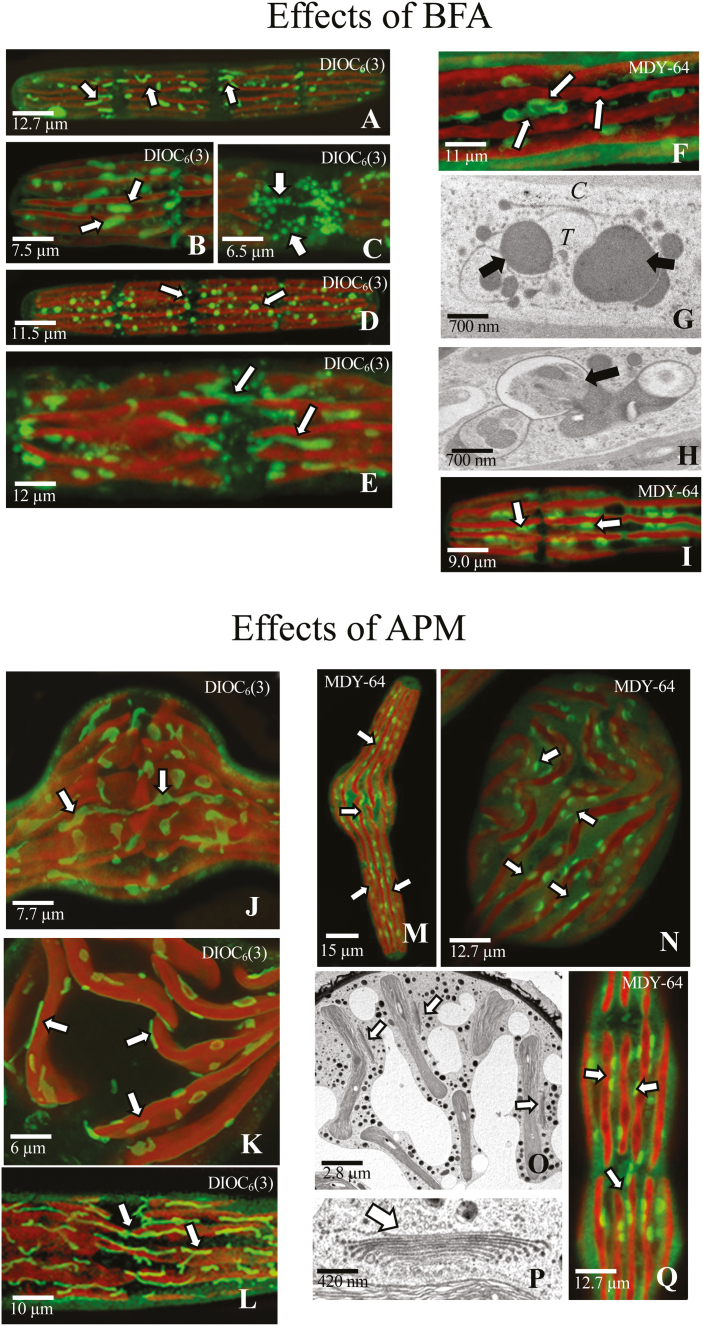
Experimental manipulation of the endomembrane system, as shown by CLSM (A–E), (I–J), (M), (N), and (Q), and TEM (G), (H), (O), and (P). (A) Disruption of the endoplasmic reticulum (ER) network after 2 h treatment with 1 μM brefeldin A (BFA), followed by DIOC_6_(3) labeling. The elongate tubules transformed into short segments (arrows). (B) Magnified view of the flattened segments of ER (arrows) in cells treated with 1 μM BFA for 2 h, followed by DIOC_6_(3) labeling. (C) 4 h of treatment with BFA resulted in complete disruption of the ER into small circular segments (arrows), visualized by DIOC_6_(3). (D) BFA treatment for 6 h resulted in small circular segments (arrows) filling the cytoplasmic valleys, visualized by DIOC_6_(3). Scale bar=12 μm. (E) After 4 h of recovery, the tubular elements of the ER began to reform (arrows), visualized by DIOC_6_(3). Scale bar=5.8 μm. (F) MDY-64 labeling of a cell treated with BFA for 2 h showing Golgi bodies transformed into irregular, curled entities (arrows). Scale bar=11 μm. (G) Golgi body in a cell treated for 2 h with BFA. The *cis* face (*C*) consisted of a few cisternae and subtended a network of cisternae/large vacuoles (arrows) at the *trans* face (*T*). Scale bar=700 nm. (H) Unusual membranous mass (arrow) found in the subcortical cytoplasm after a 2 h BFA treatment. Scale bar=700 nm. (I) Golgi bodies returning to a normal morphology and positioning in the cytoplasmic valleys after 2 h of recovery, visualized by MDY-64 (arrows). Scale bar=9.1 μm. (J) Disruption of the ER network into short segments of flattened tubules (arrows) in cells treated with APM for 12 h, visualized by DIOC_6_(3). Scale bar=7.7 μm. (K) Magnified view of altered ER segments (arrows) showing close proximity to the chloroplast envelope in a cell treated with APM for 24 h, visualized by DIOC_6_(3). Scale bar=6 μm. (L) Long ER tubules (arrows) reformed after 12 h of recovery, visualized by DIOC_6_(3). Scale bar=10 μm. (M) Swelling at the isthmus and Golgi bodies (green) still positioned in the cytoplasmic valleys after 12 h APM treatment, visualized by MDY-64. Scale bar=15 μm. (N) Irregularly positioned Golgi bodies (green) in the swollen isthmus zone after APM treatment for 24 h. Scale bar=12.75 μm. (O) Golgi body structure (arrowhead) showing close proximity to the chloroplast lobes in the swollen isthmus after APM treatment. Scale bar=2.8 μm. (P) Golgi body (arrowhead) showing similar architecture to untreated cells after APM treatment. Scale bar=420 nm. (Q) After 8 h of recovery, the cell reformed into its cylindrical shape and Golgi bodies position in the cytoplasmic valleys. Scale bar=12.7 μm.

In order to resolve the structural dynamics of the endomembrane system in live cells, we incubated them in medium containing several subcellular-disrupting agents and monitored changes using DIOC_6_(3) and MDY-64 labeling. The agents tested include the Golgi body endocytosis-affecting agent, BFA, the microtubule-altering compound, APM, and actin microfilament-altering agents, CB and LatB. We also monitored changes in cells placed under physical stress, including high light and desiccation conditions. Three notable morphological alterations are observed under these experimental treatments (Supplementary Fig. S1A–G). First, treatments with BFA result in a notable decrease in cell length, by 30±2%. Secondly, treatment with APM causes significant swelling at the isthmus. Thirdly, ‘filamentous’ phenotypes are generated in cells treated with CB or LatB. After removal of the experimental agents by repetitive washing and a return to culture (i.e. recovery), the cylindrical unicell phenotype reappears in post-cell division progeny (i.e. 24–72 h). We also monitored cell wall expansion in cells under various treatments by labeling with an mAb, JIM5, which recognizes the pectin polymer homogalacturonan, and subsequently monitoring new wall growth (Supplementary Fig. S1H–N). Only cells treated with BFA do not expand during treatment, and this effect is also reversible upon recovery. To monitor EPS secretion, we labeled cells with Fluoresbrite fluorescent polyspheres (Supplementary Fig. S1O, P). Again, only BFA treatment resulted in inhibition of EPS secretion. When cells are treated with 1 μg ml^–1^ BFA for 2 h, the ER disperses into short tubular segments (Fig. 6A, B) and after 24 h the ER tubules further transformed into spherical bodies (Fig. 6C, D) that remain in the cytoplasmic valleys. When cells were washed free of BFA and allowed to recover, the ER begins to reform within 1 h (Fig. 6E) and ultimately yields the typical series of long tubules. When cells were incubated for 2 h in BFA and then labeled with MDY-64, Golgi bodies transform into irregular membranous swirls ranging in size from 2 μm to 4 μm (Fig. 6F). TEM examination of BFA-treated cells shows that individual Golgi bodies are significantly altered and consist of a small stack of cisternae, most probably remnants of the *cis* face cisternae, which are loosely associated with an aggregation of irregular large osmiophilic vesicles/vacuoles located at the former *trans* face (Fig. 6G). The peripheral cytoplasm also contains swollen bodies, comprised of circular cisternae and vacuole-like structures (Fig. 6H). Upon recovery for 24 h, the Golgi bodies reappeared (Fig. 6I).

**Fig. 7.**
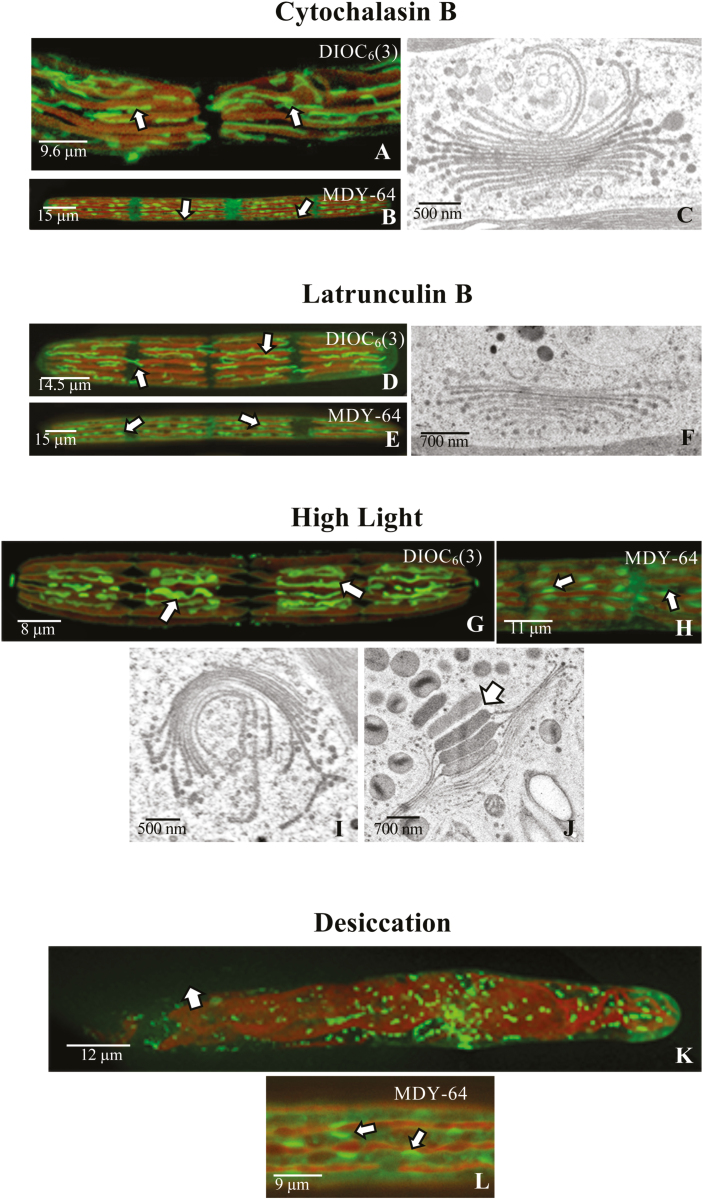
Experimental manipulation of the endomembrane system as shown by CLSM (A), (B), (D), (E), (G), (H), (K), and (L), and TEM (C), (F), (I), and (J). (A) Upon treatment with 5 μg ml^–1^ CB for 24 h, the endoplasmic reticulum (ER) network of long tubules (arrows) remains, as visualized by DIOC_6_(3). (B) CB treatment does not alter the morphology or positioning of Golgi bodies (arrows), visualized by MDY-64. (C) TEM image of a Golgi body from a cell treated with CB. (D) Treatment with 5 μM LatB did not result in a notably disturbed ER tubule network (arrows), visualized by DIOC_6_(3). (E) LatB treatment did not alter the positioning of Golgi bodies (arrows), visualized by MDY-64. (F) Unaltered Golgi body from a LatB-treated cell. (G) Flattened ER (arrows), which is absent in the vacuolar zone between the chloroplast and at the isthmus, in cells grown under high light, visualized by DIOC_6_(3). The positioning of parallel ER tubes is maintained. (H) High light treatment does not alter the positioning of the Golgi bodies (arrows) as shown by MDY-64 labeling. (I) Golgi body under high light conditions, showing notable curling but general preservation of architecture. (J) Golgi body under high light conditions, showing large swellings that yield extracellular polysaccharide (EPS) vesicles (arrows). (K) Under desiccation conditions, the ER network (arrows) transforms into small segments as seen using DIOC_6_(3). (L) Under desiccation conditions, the Golgi bodies (arrows) remained in the cytoplasmic valleys, visualized by MDY-64

When cells were incubated with 1 μM APM, the isthmus region (i.e. the site of cell wall expansion) swells and the chloroplast lobing became irregular. Within 12 h, the ER disperses into short plate-like sheets (Fig. 6J), remaining in close proximity to the chloroplast membrane (Fig. 6K). When cells were allowed to recover, the cylindrical cell phenotype returns, the chloroplast reforms its lobed structure, and the long ER tubules reappear (Fig. 6L). When cells were treated with APM for 12 h and then labeled with MDY-64, the long rows of Golgi bodies remain intact throughout most of the cell (Fig 6M) but are irregularly dispersed in the swollen isthmus (Fig. 6N). This displacement is most likely to be due to the altered distribution of cytoplasmic valleys caused by the significant chloroplast transformation during swelling of the isthmus. TEM analysis shows that Golgi body structure is not altered (Fig. 6O, P). Upon recovery, the cylindrical phenotype returns within 48 h, along with the organized linear rows of Golgi bodies (Fig. 6Q). Treatment of cells with the actin-disrupting agents CB and LatB had no effect on the structure or positioning of the ER and Golgi bodies (Fig. 7A–F). However, high light treatment resulted in subtle changes to the endomembrane components and, while Golgi body distribution appeared unchanged, the long ER tubules are absent from the large vacuolate regions of the treated cells as well as the isthmus (Fig. 7G–J). Under desiccation treatment, chloroplast morphology changed due to the production of large amounts of starch granules. This results in the formation and displacement of the ER tubules (Fig. 7K), but Golgi body distribution remains similar to that observed in untreated cells (Fig. 7L).

### BLAST search and gene comparisons

**Fig. 8.**
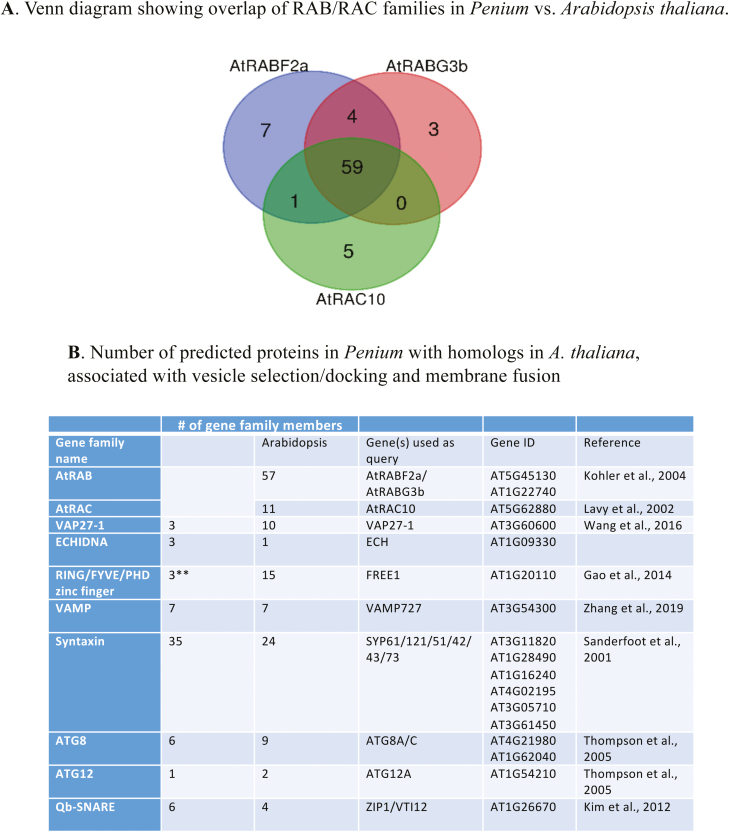
(A) Venn diagram showing the number of hits and their overlap when searching the *Penium* protein database using AtRABF2a, AtRABG3b, or AtRAC10 as queries. The diagram was made using the online tool at: http://bioinformatics.psb.ugent.be/webtools/Venn/. (B) Summary of the number of predicted proteins in *Penium* with homologs in *A. thaliana*, associated with vesicle selection/docking and membrane fusion. *Penium* homologs in *A. thaliana* (AT) were identified based on queries in a local BLAST search (<1e-5) of GenBank (www.ncbi.nlm.nih.gov/genbank/). * indicates an overlap in the retrieved gene lists. ** indicates a cut-off of <1e-15, used due to the presence of a high proportion of repetitive sequence. The last column gives literature references for the number of members in the *A. thaliana* family.

When comparing the RAB/RAC families of Arabidopsis and *Penium*, there is overlap in the retrieved gene lists as shown in the Venn diagram in Fig. 8A; however, the total number of genes is similar to that in *A. thaliana*. Several key proteins involved in vesicle selection/docking and membrane fusion in Arabidopsis are also found in *Penium* (Fig. 8B).

## Discussion

### The geographic, structural, and functional dynamics of Golgi bodies in *Penium*

Many zygnemataphycean algae possess an ECM that is often produced in large quantities, especially in transient blooms that appear in ephemeral ponds and shallow wetlands that frequently experience desiccation (Boney, 1981; Brook, 1981; Oertel *et al.*, 2004; Domozych *et al.*, 2005; Domozych and Domozych, 2008; Wehr *et al.*, 2015). The ancestors of these algae also frequented shallow freshwater wetlands ~500 million years ago (Delwiche and Cooper, 2015), and very probably used similar ECM components to survive in these rapidly changing aquatic–terrestrial interface ecosystems, as well as for colonizing land.

**Fig. 9.**
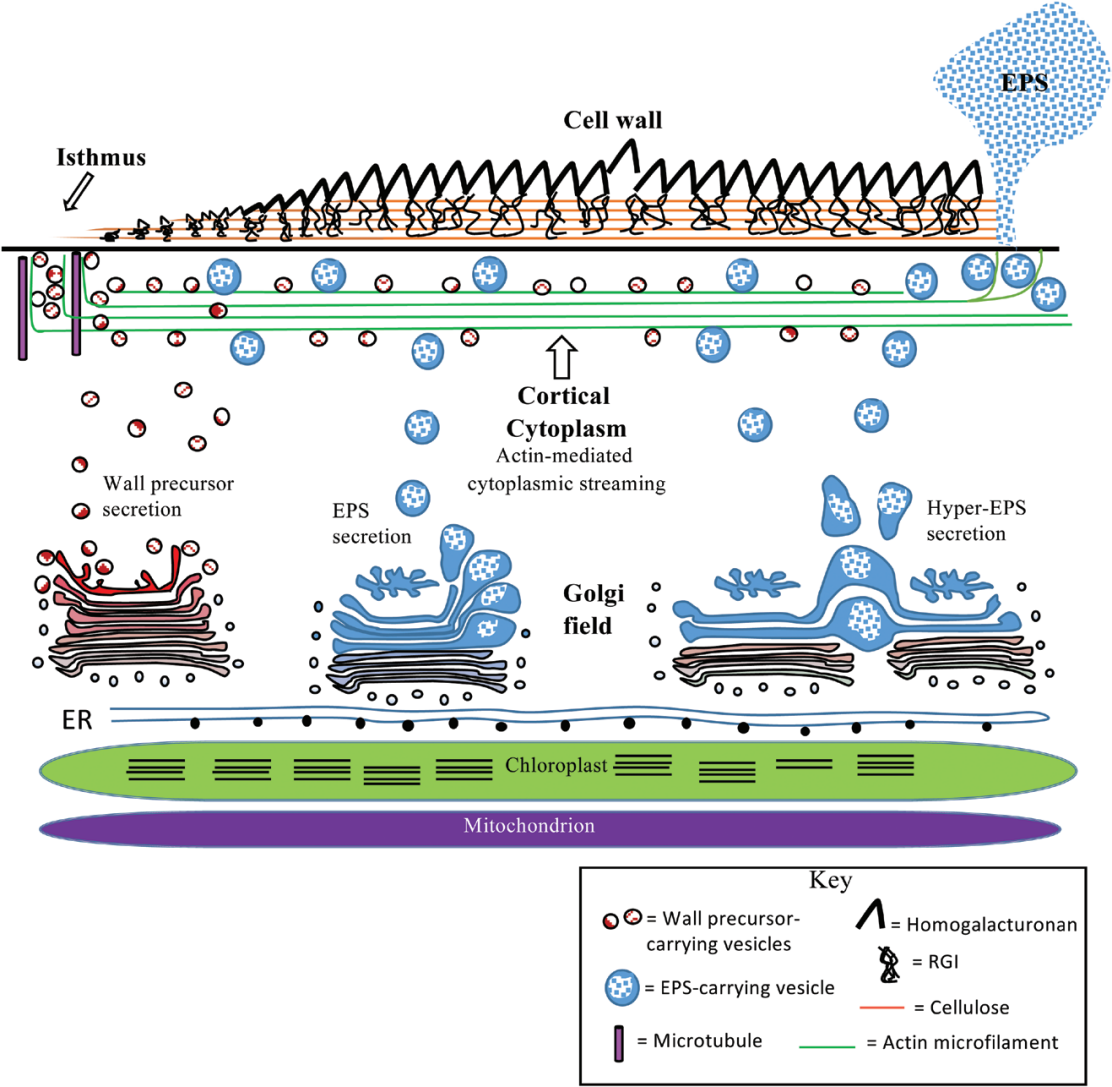
Model of the secretory apparatus of *Penium*. A total of 150–200 Golgi bodies align in linear fields deep in the cytoplasmic valleys created by the chloroplast lobes. The Golgi bodies sit above endoplasmic reticulum (ER) that is closely associated with the chloroplast envelope. Elongate mitochondria are also present in this valley. Based on the immunogold labeling of this study, we believe that some Golgi bodies process wall precursors while others process the EPS. In EPS-producing Golgi bodies, EPS vesicle formation appears on only one side of the cisternal stack. In hypersecretion phases (e.g. cells grown under high light), Golgi bodies appear fused. Golgi-derived vesicles move to the cortical cytoplasm where they are transported around the cell via cytoplasmic streaming. The cortical cytoplasm contains large amounts of actin microfilament bundles. Wall precursor vesicles are ultimately delivered to the isthmus zone where bands of microtubules and microfilaments line the expansion zone. EPS vesicles are sent to various regions of the cell periphery for subsequent EPS release. These zones are transient and change in response to environmental cues.

The biosynthetic and secretory machinery that is required to produce the diverse and quantitatively large amounts of ECM in these zygnematophycean algae is extensive, highly organized, and one that rapidly responds to developmental cues and external stresses. We observed this in the architectural design of the endomembrane system of *Penium* that is highlighted by 150–200 individual Golgi bodies. The Golgi bodies are juxtaposed to each other in linear arrays that are oriented parallel to the long axis of the cell (Figs 1K, 2C). This arrangement is distinct from that of land plants, where the Golgi bodies are present in a polydisperse configuration, and from animal cells where the Golgi bodies are found in perinuclear ribbons (Robinson *et al.*, 2015). This large number of Golgi bodies per cell and their close if not ‘fixed’ positioning near the ER, chloroplast, and mitochondria are also common characteristics of other unicellular zygematophyceaen taxa (Ueda and Noguchi, 1997). We propose that the organized layering of the endomembrane system and other organelles provides for a highly efficient secretory apparatus, with the ECM biosynthetic and packaging machinery optimally positioned near its chief sources of photosynthate and energy (i.e. chloroplast and mitochondria). The large number of Golgi bodies also highlights a secretory system capable of rapid production of large amounts of ECM cargo. A putative model describing the organization of the endomembrane system and the secretory apparatus is provided in Fig. 9.

Each Golgi body displays distinct *cis*, medial, and *trans* loci (Figs 1L, J, 2D). A pool of small vesicles fills the zone between the *cis* face and underlying ER that probably deliver cargo to the Golgi body from the ER (e.g. COPII vesicles). The Golgi body periphery and TGN are enveloped by numerous small vesicles, some of which carry wall precursors to the cortical cytoplasm. In many Golgi bodies, the peripheries of medial–*trans* face cisternae produce, and are then surrounded by, large vesicles that carry EPS. Interestingly, the EPS-containing cisternal swellings and EPS vesicles are observed only at one side of the Golgi body (Fig. 3F). These observations were supported by our tomography analysis and correspond to a similar Golgi profile reported in an earlier study of the desmid, *Netrium* (Eder and Lütz-Meindl, 2010). This indicates that distinct geographic locations exist in a single Golgi body for processing of different ECM components. This would provide a direct means to separate the different biosynthetic machineries that produce the diverse and highly complex polysaccharides and proteoglycans found in the ECM. Such an organization would presumably also allow faster and more efficient processing of EPS during periods when delivery of a large amount of EPS to the cortical cytoplasm is required. Additionally, we noted that not all Golgi bodies appear to produce EPS vesicles at any one time, suggesting a spatiotemporal differentiation of Golgi bodies for different ECM-processing activities. Further study will be needed to determine whether EPS-producing Golgi bodies can process cell wall precursors under specific conditions.

Another notable observation was the direct connection of the cisternal peripheries of adjacent Golgi bodies processing EPS (Fig. 3G, H). Do these Golgi complexes represent stages in the fission of Golgi bodies or are they a product of Golgi body fusion? The first possibility implies that these Golgi body profiles simply represent stages in Golgi body division occurring at times when the cell is also secreting large amounts of EPS. However, it is also feasible that the cisternal peripheries of closely packed Golgi bodies make physical contact and ultimately fuse with each other during EPS processing. Notably, we observed that medial–*trans* cisternae of adjacent Golgi bodies at the same stage of EPS processing, and with very similar membrane architecture, fuse and create ‘hybrid’ Golgi complexes. Golgi body fusion is a phenomenon that has been reported before in other green algae (Ueda and Noguchi, 1997; Hummel *et al.*, 2007). In *Penium*, the fused Golgi complexes may facilitate and/or enhance EPS packaging and secretion during hyper*-*EPS secretion periods. For example, we commonly noted these complexes in cells growing under high light treatment, a condition that stimulates EPS production.

Our immunogold studies (Fig. 5) revealed that epitopes of the cell wall components RG-I and AGP are processed in medial–*trans* face cisternae and the TGN of the same Golgi body. This was demonstrated in both single and co-labeling experiments, and shows that some Golgi bodies process multiple cell wall precursors simultaneously. EPS labeling was found in the large swellings on, and vesicles derived from, the cisternal peripheries of the *trans* face. We saw no evidence that RG-I or AGP epitopes co-localize with EPS in the same Golgi body. This supports the aforementioned idea that EPS and cell wall components are processed in different Golgi bodies. Golgi-derived vesicles only labeled for single epitopes (i.e. no co-labeling) of RG-I, AGP, or EPS. This indicates that specific ECM components are segregated and packaged in specific vesicles before delivery to the cortical cytoplasm.

### Secretion dynamics: post-Golgi delivery of extracellular components

In *Penium*, the Golgi bodies are not mobile. This feature is notably different from what has been described in cells of several land plant taxa, where Golgi bodies are mobile and dock onto the ER prior to processing materials for subsequent secretion (e.g. ‘mobile secretory units’; Osterrieder *et al.*, 2017; see also Boevink *et al.*, 1998; Nebenfuhr and Staehelin, 2001; Hawes and Satiat-Jeunemaitre, 2005; Woodhouse and Goldstein, 2013; Hawes *et al.*, 2015; Ueda *et al.*, 2010; Sparkes *et al*., 2011, 2009; Kim and Brandizzi, 2016; Stefano and Brandizzi, 2018). Cytoplasmic streaming in *Penium* does not occur in the cytoplasmic valleys that contain the Golgi bodies and ER. Rather, ECM vesicles arising from stationary Golgi bodies in these valleys move to a thin layer of cortical cytoplasm where they are transported around the cell by actin-mediated cytoplasmic streaming. Vesicles then exit from the streaming cytoplasm at specific sites of the cell periphery, fuse with the plasma membrane, and secrete their cargo. The ECM secretory apparatus of *Penium* is therefore based on producing and maintaining a ready supply of mobile ECM vesicles, not Golgi bodies, near potential secretion sites. The narrow cortical cytoplasmic streaming zone represents a mobile ‘highway’ that carries diverse ECM-containing vesicle traffic in close proximity to plasma membrane fusion sites. This type of ECM cargo delivery system allows for rapid delivery of ECM components to specific secretion sites, especially when these sites change in response to environmental or developmental signals. For example, *Penium* cell gliding requires rapid secretion of large amounts of EPS from one polar zone of the cell (Domozych *et al.*, 2005; Domozych and Domozych, 2008). However, the EPS secretion site may then quickly change to another part of the cell. The presence of a large and mobile population of EPS vesicles in the cortical cytoplasm would provide an immediate source of EPS for these transient EPS secretion sites.

The vesicle traffic transported on the cortical cytoplasmic highway is diverse (i.e. carrying various cell wall and EPS cargo) and requires ‘exit ramps’ in order to deliver different ECM cargo to precisely defined plasma membrane fusion sites. What might constitute these highly specific exit ramps? First, sets of vesicle selection/docking proteins and membrane fusion proteins that have been characterized in land plants and other eukaryotes (e.g. SNAREs, RAB GTPases; Ebine and Ueda, 2015; Marais *et al.*, 2015; Barlow and Dacks 2018), and that identify specific vesicles, would be critical components of such exit ramps. Our BLAST analysis also provided evidence for the existence of key proteins of the secretory mechanism of *Penium* (Fig. 8). Characterization of these proteins will be the target of future studies. Secondly, a network of cortical actin microfilaments (Ochs *et al.*, 2014) must also be critical for this mechanism. For example, while microfilament bundles of the cortical cytoplasm may be responsible for general transport of vesicles around the cell, it could be that single or small aggregates of microfilaments branch off the bundles and direct vesicle traffic to specific plasma membrane fusion zones. These microfilament-defined exit ramps would be capable of rapidly forming along the cortical cytoplasm–plasma membrane interface in response to external signals or internal cues. This would be similar to phenomena observed in other plant cells (Vidali *et al.*, 2009; Fu, 2015; Mendrinna and Persson, 2015; Grebnev *et al.*, 2017; Jiang *et al.*, 2019).

Cell wall secretion dynamics in *Penium* are further compounded by temporal control during cell development. For example, previous work (Domozych *et al.*, 2014) has demonstrated that during cell wall expansion preceding cell division, cellulose and RG-I are the first components deposited at the isthmus. This is followed by the deposition of the HG that forms a distinctive cell wall lattice. This ordered development of the cell wall is probably controlled by sequential secretion/deposition of cell wall precursors and subsequent interpolymeric interactions in the isthmus apoplast. However, precisely regulated synthesis and delivery of wall precursors via the endomembrane system must also be involved in this process. Specific sets of docking/fusion proteins that only recognize specific wall precursor-containing vesicles are most probably located at the isthmus and facilitate fusion with the plasma membrane. These proteins would be functional only during specific cell cycle phases. Again, the cytoskeleton of the isthmus must also play a key role in directing specific vesicles to the isthmus zone. Ochs *et al.* (2014) described distinct cortical bands of microtubules and microfilaments that encircle the isthmus zone. These may serve as collection centers for vesicles carrying cell wall cargo and interact with plasma membrane proteins to control vesicle fusion with the plasma membrane and secretion of their constituents. This would be similar to the networks of actin microfilaments and microtubules that have been shown to be directly involved in regulating vesicular traffic during cell wall deposition and expansion in land plants (Verchot-Lubicz and Goldstein, 2010; Dong *et al.*, 2012; Sampathkumar *et al*., 2011, 2013; Bashline *et al.*, 2014; Li *et al.*, 2015; Liu *et al.*, 2015; Goring, 2017; Elliott and Shaw, 2018; Wu and Benzanilla, 2018).

It was also shown in this study that application of microfilament-disrupting agents (e.g. CB or Lat B) does not stop cell wall expansion or EPS secretion (Fig. 7A–F). The absence of an effect of these agents on ECM secretion and subsequent development is not uncommon, and has been reported in land plants (Nothnagel *et al.*, 1981; Geitmann and Nebenfuhr, 2015). Other studies have shown that movement of endomembrane components during secretion is variable and is dependent on specific configurations of actin at particular secretion sites of the cell (Akkerman *et al.*, 2011). Our observations with *Penium* also show this and demonstrate that actin microfilaments that are involved in cell wall expansion/EPS secretion act differently from those employed in cytokinesis.

### Experimental analyses of the endomembrane system

In this study, we observed MDY-64-labeled Golgi bodies in *Penium* (Figs 1K, 6F, I, M, N,Q). Coincidentally, DIOC_6_(3) has been used to label Golgi bodies of other zygnematophycean taxa, including *Micrasterias* and *Closterium* (Ueda and Noguchi, 1997), but only labeled the ER in *Penium*. While further work is necessary in order to decipher their binding activities, they represent valuable tools for studying Golgi bodies and the ER along with secretion efficacy with various experimental agents and stresses, and monitored effects with microscopy-based analyses. Of the agents/stresses tested, only BFA induced notable alterations that are similar to what has been previously described in land plant cells treated with BFA, including independent changes to both the structure and chemistry of the GA and the TGN (Robinson *et al.*, 2008; Lam *et al.*, 2009; Langhans *et al.*, 2011; Ito *et al.*, 2017). BFA inhibits the ADP ribosylation factor (ARF) and COPI and COPII vesicles, which in turn disrupts ER–Golgi vesicular transport, most notably at the *cis* face region (Moon *et al.*, 2012). BFA also produces disruption of the TGN and other endosomal membranes to form unique BFA compartments (Robinson *et al.*, 2008; Langhans *et al.*, 2011), similar to what was noted with *Penium*. Additionally, the severe effects of BFA on ER structure closely parallel the BFA-induced ER stress characteristics reported in other eukaryotes (Moon *et al.*, 2012). During BFA treatment, both cell wall expansion and EPS secretion are inhibited, but these effects are reversible. Microtubule- and microfilament-affecting agents, as well as high light and desiccation stress, did not notably affect Golgi body structure or secretion. Only minor alterations to the ER were observed. These results demonstrate the structurally robust nature of the GA and ER even under harsh experimental conditions, which probably represents a strong adaptive advantage in the native habitats of *Penium*.

### Conclusions

The extensive and highly organized endomembrane system of *Penium* is a reflection of its large and complex ECM. The secretory apparatus that it supports manufactures and deposits a highly structured cell wall that is similar in make-up to that of land plant walls. It also processes a multifunctional polysaccharide mucilage, often in prodigious amounts, that is critical to survival in shallow wetlands. Elucidation of *Penium*’s endomembrane system provides new information about plant secretion dynamics as well as insight into subcellular systems that ancient charophytes potentially employed to invade and exploit terrestrial habitats.

## Supplementary data

Supplementary data are available at *JXB* online.

**Fig. S1.** Effects of treatments on cell morphology, wall expansion, and extracellular polysaccharide secretion.

**Fig. S2.** (A) TEM image of the elongated mitochondria at the base of the cytoplasmic valleys. (B) The mitochondria also branch. (C) TEM image of EPS vesicles emerging from one side of the Golgi body but not the other. (D) Golgi body division. (E) Cortical microtubules at the isthmus that lie perpendicular to the long axis of the cell. (F) The isthmus cortical zone where small vesicle fuse with the plasma membrane. (G) Large EPS vesicles near the plasma membrane of the cortical zone near one of the cell poles.

**Fig. S3.** mAb labeling of the extracellular matrix.

eraa039_suppl_Supplementary_Figures_S1_S3

## Abbreviations

CLSM: confocal laser scanning microscopy
DIC: differential interference contrast microscopy
ECM: extracellular matrix
EPS: extracellular polysaccharide
ER: endoplasmic reticulum
FLM: fluorescence light microscopy
TGN: *trans*-Golgi network
