# Supplementary material for: Endomembrane architecture and dynamics during secretion of the extracellular matrix of the unicellular charophyte, Penium margaritaceum
Source: J Exp Bot. 2020 Feb 25;71(11):3323–39. doi: 10.1093/jxb/eraa039 (PMC7289721; doi:10.1093/jxb/eraa039)
Supplement: eraa039_suppl_Supplementary_Figures_S1_S3 [file eraa039_suppl_supplementary_figures_s1_s3.pdf]

Supplementary Figure 1

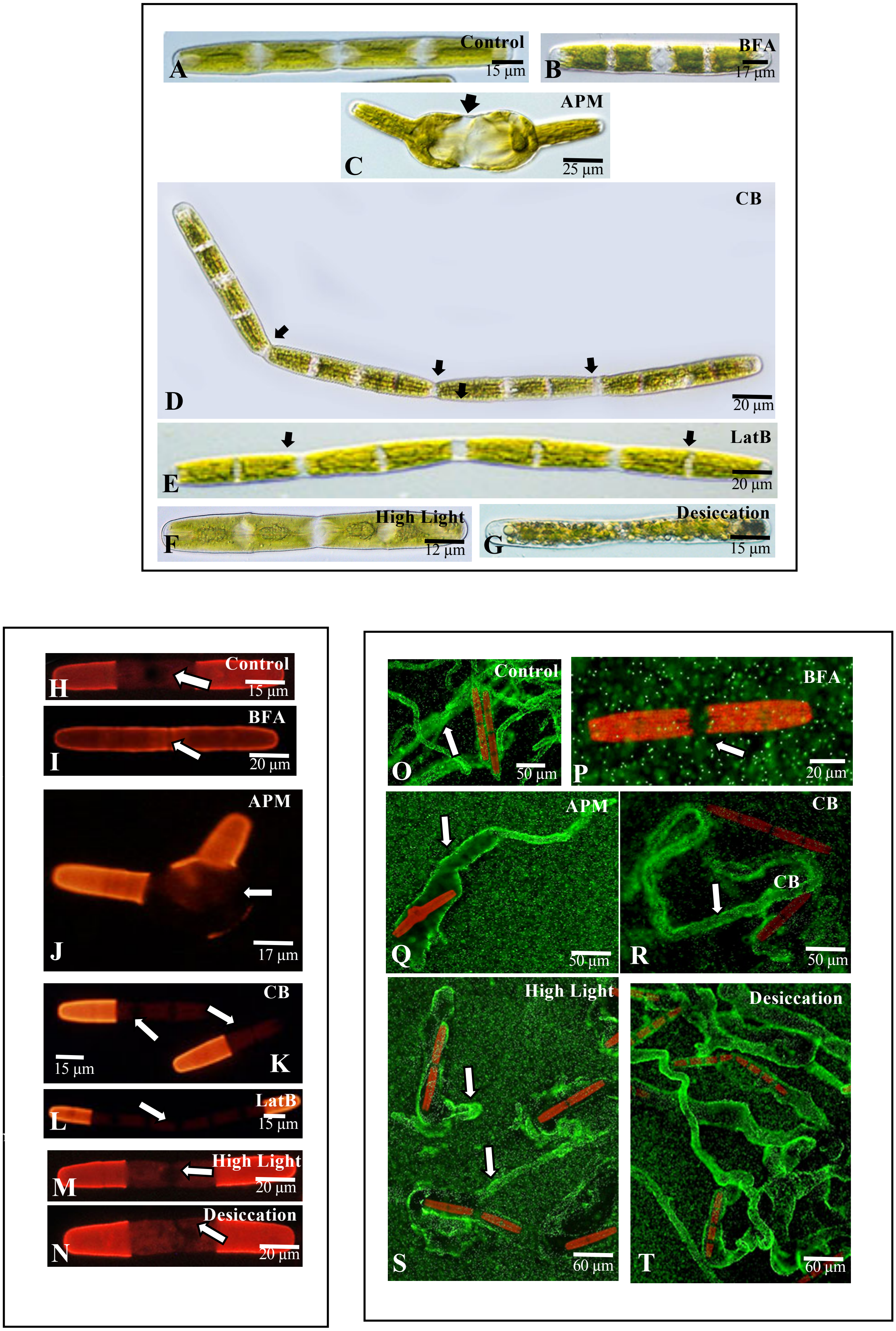

Supplementary Fig 1. Effects of treatments on cell morphology, wall expansion and extracellular polysaccharide (EPS) secretion. (A)-(G). Morphology of cells treated with various experimental agents. APM caused swelling of the isthmus area and CB (or LatB) induced formation of filamentous phenotypes. Desiccation conditions caused changes in shape and chloroplast. All other treatments do not show change. All images were taken with DIC-LM. (H)-(N**)**. Experimental effects on wall expansion. Only BFA caused a stop. All images were taken by FLM. (O)-(T**)**. Experimental effect on extracellular polysaccharide (EPS) secretion. All images were acquired by FLM. The chloroplast is highlighted by red autoflorescence.

Supplementary Figure 2

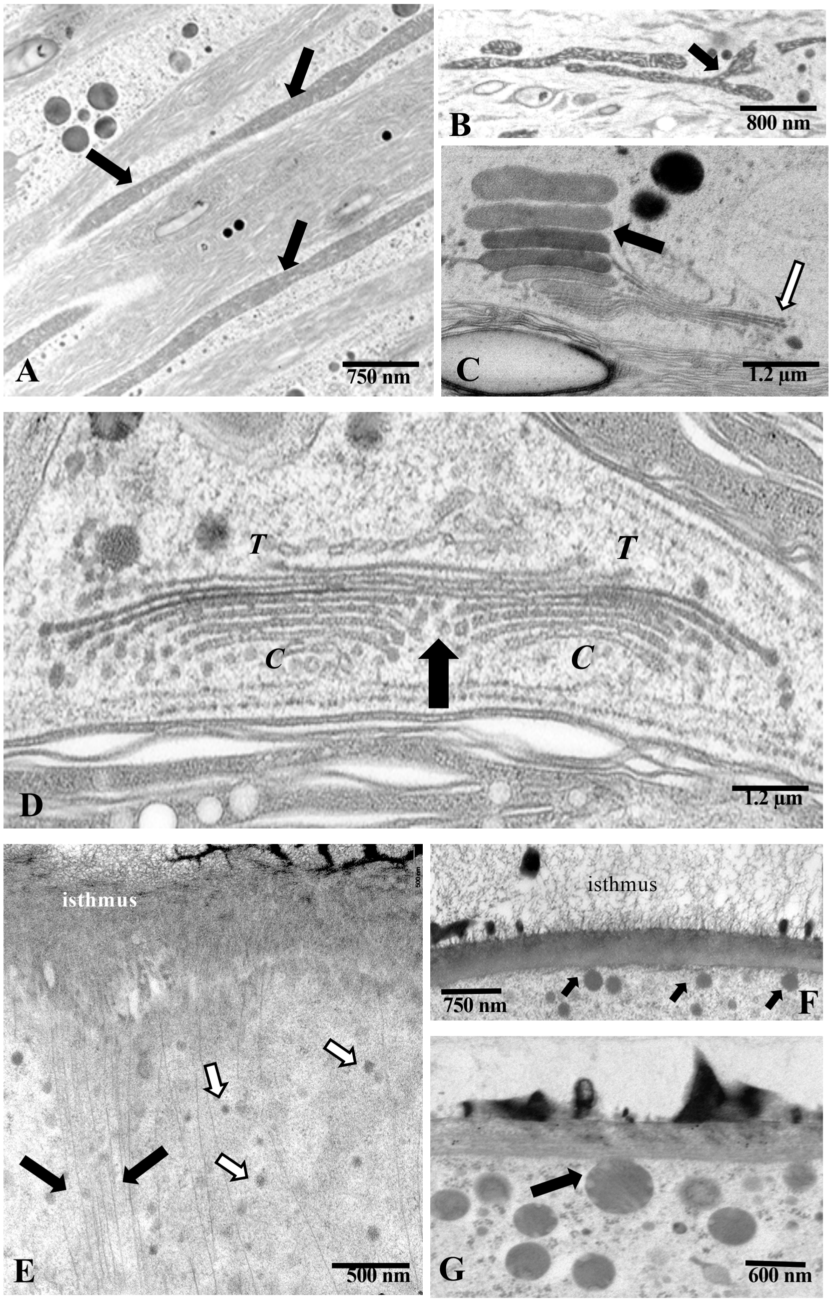

Supplementary Fig. 2. (A)TEM image of the elongated mitochondria (arrows) at the base of the cytoplasmic valleys. (B) The mitochondria also branch (arrow). (C) TEM image of extracellular polysaccharide (EPS) vesicles emerging from one side of the Golgi body (black arrow) but not the other (white arrow). (D) Golgi body division. After expansion of the Golgi, separation of the daughter Golgi bodies begins at the cis face (*C)* and terminates to the trans face (*T*). (E) Cortical microtubules at the isthmus that lie perpendicular to the long axis of the cell. The individual microtubules (black arrows) form a network where small vesicles (white vesicles) collect. (F) The isthmus cortical zone where small vesicles (arrows) fuse with the plasma membrane. (G) Large EPS vesicles (arrow) near the plasma membrane of the cortical zone near one of the cell poles.

Supplementary Fig. 3

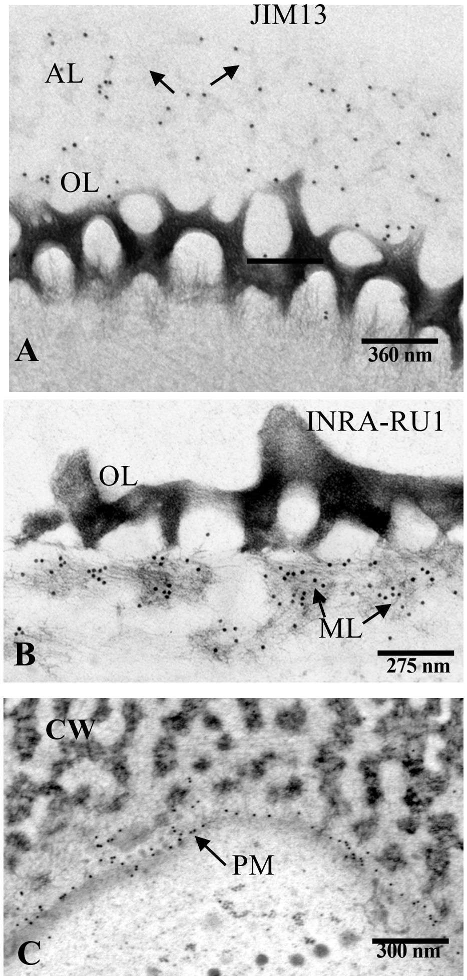

Supplementary Fig. 3. mAb labeling of the extracellular matrix (ECM). (A) JIM13 labeled an outer layer of fine fibrils outside the outer layer of the wall (OL). (B) INRA- RU1 labeled the medial layer (ML) of the cell wall. The outer pectin layer (OL) was attached to this medial layer. (C) CCRC-M80 labeled the interface of the cell wall with the plasma membrane (arrows). All images were obtained using TEM.
